# Using women advocacy groups to enhance knowledge and home management of febrile convulsion amongst mothers in a rural community of Sokoto State, Nigeria

**DOI:** 10.11604/pamj.2013.14.49.1703

**Published:** 2013-02-05

**Authors:** Oche Mansur Oche, Oloche Ben Onankpa

**Affiliations:** 1 Department of Community Medicine, Usmanu Danfodio University, Sokoto, Nigeria; 2 Department, Paediatrics, Usmanu Danfodio University, Sokoto, Nigeria

**Keywords:** Febrile convulsion, knowledge, home management, Intervention, Nigeria

## Abstract

**Introduction:**

Febrile convulsions (FC) are a common paediatric problem worldwide. Between 1 and 4% of children will have a febrile convulsion with about 4% of cases arising before the age of six months. Although FC is benign and does not cause death, brain damage or learning disorders, it is quite frightening to observers and parents who witness an episode of FC, would think the child is going to die.

**Methods:**

This was a quasi-experimental study (a pre and post-test interventional, one group), aimed at assessing the impact of health education on knowledge and home management of febrile convulsion amongst mothers in a rural community in North Western Nigeria. A one in three samples of fifty mothers that met the eligibility criteria where selected using systematic random sampling. Structured interviewer administered questionnaire with close and open-ended questions was administered to obtain data at pre- and post intervention.

**Results:**

The ages of the mothers ranged from 18-47 with a mean age of 33 ± 7.14years. The perceived causes of febrile convulsion included fever (28%), witch craft (80%) with majority (98%) of the mothers administering traditional medications. Proportion of study subjects with adequate knowledge of febrile convulsion at baseline and post intervention were 4% (mean knowledge score of 35.3± 9.48) and 96.0% (mean knowledge score of 77.69 ± 10.75) respectively (P < 0.0001).

**Conclusion:**

Although inadequate knowledge and inappropriate home practices about FC were rampant in the study community, using community members to teach and sensitize the mothers on FC improved their knowledge base significantly. The use of effective educational intervention programmes and parental support groups will go a long way in reducing the incidence of FC among children in our communities.

## Introduction

In 1980, a consensus conference held by the National Institute of Health described febrile convulsion (FC) as, “An event in infancy or childhood usually occurring between three months and five years of age, associated with fever, but without evidence of intracranial infection or defined cause [1]. Any illness that causes a fever (high temperature) can cause FC. Most occur with common illnesses such as ear infections, coughs, colds, flu and other viral infections. Serious infections such as pneumonia, kidney infections, meningitis, etc, are less common causes [2] but in the tropics, malaria is a major cause of FC.

Seizures are generally referred to as *simple* if they last less than 15 minutes, do not re-occur within 24 hours and are generalized; the tonic-clonic contractions involve the head, spine and all four limbs. In contrast, *complex seizures* last for more than 15 minutes, reoccur within 24 hours, or have a partial (focal) onset, according to criteria of the International League against Epilepsy [3].

Epidemiology of the disease shows that between 2 and 4% of children will have a febrile convulsion and about 4% of cases arise before 6 months old, 90% between 6 months and 3 years and the other 6% over three years. Also between 2 and 5% of children experience at least one FC before the age of 5 years [4]. The natural history, potential risks of recurrence, subsequent development of epilepsy and prophylactic medications have become well understood over the years [5–9]. Although FC is benign and does not cause death, brain damage or learning disorders, it is quite frightening to observers and parents who witness an episode of FC, would think the child is going to die [10, 11].

The lack of proper knowledge of home management of FC by mothers has resulted in panic and confusion necessitating the use of various unorthodox remedies in various parts of Nigeria including the use of human or cow's urine, kerosene, palm oil and bloodletting with razor blade to treat FC at home. Physicians are often quick to use medications to treat this rather benign disorder without proper education and counselling as a result of their misunderstanding of the consequences of seizure recurrence [6, 12, 13]. The best approach for FC should involve establishment of a good communication with parents and should be aimed at improving their responses to seizures at home hence it is the parents, rather than the FC children who need treatment [14]. It is of particular importance that the families are relieved of their concerns and are capable of intervening optimally with the disease [12, 15]

Several studies have investigated the aetiology and natural history of FC and evaluated various management strategies, but little or no information is available about the proper home management of this disorder in the study area. It is in realisation of the high morbidity and mortality associated with FC in developing countries including Nigeria and lack of proper knowledge of home management of this disorder that it became imperative to carry out this study. This study is therefore aimed at improving the knowledge and home management of FC among mothers in the study area.

## Methods

Garabshi is a peasant settlement in Wamakko local government area of Sokoto State, North Western Nigeria with a population of 776 [16]. The indigenous inhabitants of the study area are mainly Hausas and Fulanis. The study was quasi experimental using the one group pre- and post-test design with the study population consisting of women of child bearing age (WCBA). Mothers with at least a child or children aged 6 months to 6 years were eligible for the study. Advocacy visit was paid to the community leader and elders where the objective of the study was explained and their cooperation solicited in identifying women advocacy groups within the community. These women advocacy groups had before now been used to sensitize the community and enhance such programmes as the routine immunizations, immunization plus days (IPDs) and women literacy campaigns. Eight members of the group were identified to serve as “avoid febrile convulsion advocates” and saddled with the responsibility of identifying WCBA in the households and also disseminating the necessary information about FC to the mothers. There are 312 houses in Garabshi with a total population of women of child bearing age of 170. Using systematic sampling method, a one in three samples of fifty mothers was recruited for the study. In a house with no woman of child bearing age with eligible children, the next house was chosen and since polygamy is well entrenched in the community, in a house with more than one woman of child bearing age, one was chosen by using simple random sampling using a toss of coin. Verbal informed consent of heads of households and respondents was obtained prior to the commencement of the study.

A set of structured interviewer administered questionnaires with close and open-ended questions was applied to obtain information from the mothers by five research assistants who were earlier trained on the objectives of the study, instruments of data collection and interpersonal communication. The questionnaire comprised of three sections namely socio-demographic characteristics, knowledge of FC and home practices with regards to management of FC and was pretested in another community in a separate local government area. A day was set aside after the collection of baseline date from the respondents for the training of the women advocates, the training being basically health education on the causes, myths and misperceptions and proper home management of febrile convulsion. The training was conducted for two days, lasted for two hours and was rounded off with a question and answer session and demonstrations of how to manage febrile convulsion at home. After the training, eight neighbourhoods were created with one advocate being in charge of a neighbourhood. The advocates in turn trains the respondents on what to do when their children have febrile convulsion and thereafter pays regular visits to the homes of the respondents to reinforce the knowledge they had acquired earlier. Three months after collection of the baseline data from the respondents, the same set of questionnaire was applied to the respondents to obtain the post intervention data.

Each correct answer to a knowledge question attracted one mark with no marks awarded to wrong answers. Knowledge scores less than 50 and greater or equal to 50% were adjudged inadequate and adequate knowledge respectively. Practice (home management) was graded as either appropriate or inappropriate. The appropriate methods of home management of febrile convulsion include removal of cloths if any, tepid sponging, lying the child on his or her side, keep calm and taking the child to hospital), scores less than 50 and greater or equal to 50% were adjudged inappropriate and appropriate practices respectively.

The data were entered into and analyzed using EPI-Info version 3.4.2 computer soft ware 2008. All the statistical analysis was set at 5% level of significance (i.e. 95% confidence level). Differences in parental knowledge and practice with regards to FC were tested by chi square and t-tests.

## Results

A total of fifty mothers took part in the study with a 100% response rate. The ages of the mothers ranged from 18-47 years with a mean age of 33±7.14. Forty four (88%) of the respondents were married, 4(8%) divorcees, while the rest 2(4%) were widows. All the study subjects 50(100%) had no formal (Western) education but, all had Quranic education before marriage and were all of Islamic religious background 50 (100%). Six (12%) of the respondents were petty traders, while the rest were unemployed full time house wives.

Thirty eight (76%) of the respondents were aware of febrile convulsion, with mothers being the commonest source of information 15(57%) (Table 1). At baseline, the perceived causes of febrile convulsion included fever 14(28%), witchcraft 40(80%) and bad air 49(98%) (Table 2). The various home practices of febrile convulsion at pre-intervention included removal of cloths 12(24%), take to hospital 12(24%), tepid sponging 14(28%) and administering of traditional medications 49(98%)(Table 3). Also, only a small proportion, 4% of the study subjects had adequate knowledge of febrile convulsion and its causes with a mean knowledge score of 35.3± 9.48. A total of 8(16%) of study subjects had appropriate practice of febrile convulsion.

**Table 1:** Source of information on febrile convulsion (n=38)

Source	n(%)
Mother	15(40)
Neighbour	10(26)
Hospital staff	6(16)
Friends	4(11)
Others	3(8)

**Table 2:** Perceived causes of febrile convulsion

	Pre-intervention	Post-intervention	
Causes of febrile convulsion	n(%)	n(%)	Test statistics
Fever	14(28)	33(66)	X^2^=104.10;df = 5 P < 0.0001
Witch craft	40(80)	2(4)	
Bad air	49((98)	3(6)	
Bad water	27(54)	2(4)	
Evil spirit	48(96)	4(8)	
Bad blood	47(94)	6(12)	

**Table 3:** Home practices during febrile convulsion

Home practices	Pre-intervention	Post-intervention	Test Statistics
	n (%)	n (%)	
Tepid sponging	14(28)	46(92)	X^2^=196.7;df = 7 P < 0.0001
Scarification marks	45(90)	2(4)	
Traditional Medication	49(98)	3(6)	
Spoon in the mouth	37(74)	3(6)	
Holding/restriction	37(74)	2(4)	
Remove clothes	12(24)	47(94)	
Take to hospital	12(24)	47(94)	
Cow urine	2(4)	0( 0)	

*Multiple answers allowed

Following the intervention, the proportion of respondents who were aware of febrile convulsion increased to 96% with only one (2%) of the respondents saying febrile convulsion was communicable. On their perception of the cause of febrile convulsion, the majority, 46(92%) of the respondents believed fever to be the major cause of febrile convulsion (92%) while only two of them attributed it to witchcraft (Table 2). The difference in the perceptions of the mothers with regards to the causes of FC pre and post intervention was found to be statistically significant (P<0.0001).

With respect to what the mothers do during episodes of febrile convulsion, 92 and 94% of the respondents agreed to removal of cloths and taking the child to hospital respectively (Table 3). Also following intervention, the proportion of the respondents with adequate knowledge of febrile convulsion increased from 4 to 92% with a mean knowledge score of 77.09 ± 10.75 and this was found to be statistically significant (PTable 4).

**Table 4:** Knowledge and home management of febrile convulsion

Knowledge	Pre-intervention n(%)	Post-intervention n(%)	Test statistics
Adequate	2(4)	46(92)	P < 0.0001
Inadequate	48(96)	4(8)	
Mean knowledge score	35.3 ±9.5	77.09±10.8	t = 20.6;df = 98 p < 0.0001
Home management of FC			
Appropriate	8(16)	47(94)	P < 0.0001
Inappropriate	42(84)	3(6)	

**Table 5:** Questions used to assess knowledge of febrile convulsion

Questions	Correct answer (True T, False F)
FC is same as epilepsy	F
FC is life threatening	F
FC is communicable	F
FC is curable	F
Fever can cause FC	T
FC can cause brain damage	F
Anticonvusants are required for every FC child	F
FC is caused by evil spirits	F
It is shameful to have an FC child	F
Parents should take the temperature of children with FC frequently	F

FC : febrile convulsion

## Discussion

Febrile convulsions are a common paediatric problem, they can be extremely frightening, emotionally traumatic and anxiety provoking when witnessed by parents for fear of death. However, febrile convulsions carry a good prognosis around the world but, associated with a high morbidity and mortality in Africa. Several studies had in the past only elicited the reactions and feelings of parents during episodes of FC. Health care providers need to understand the beliefs and behaviours of parents whose children have FC in order to ensure the institution of optimal and appropriate care, thus limiting morbidity, mortality and disability.

Maternal education is related to knowledge of good child care practices and household wealth. In our study, all the mothers had no western education; this is in consonance with Nigeria Demographic and health survey that showed North Western Nigeria where the study area is situated to be one of the regions with the highest proportion of women with no formal education, roughly seven in ten women. Also the North western region has the lowest female school enrolment and attendance rate (37.1%) in Nigeria [17].

There was a generally low level of knowledge about FC as only 10% of the mothers had adequate knowledge at baseline. Only 28% of our study subjects believed fever was the cause of FC. This is in consonance with the findings from the works of Ofovwe and his colleagues in Benin city, Nigeria where only 25% of their study subjects attributed the cause of FC to fever [18]. However, studies from India and Turkey, observed high proportions of their subjects (91.4% and 53% respectively) who attributed the cause of FC to fever [19, 20]. The low knowledge of FC recorded in our study may not be unrelated to the fact the our study area was a typical rural setting in Nigeria and also the fact that all our respondents were illiterates which is typical of almost all rural areas in Nigeria where the literacy level is very low [17]. Almost all our study subjects (96%) also attributed the cause of FC to witchcraft and evil spirits. Similar findings have been observed in other studies [18, 20]. These erroneous beliefs about the cause of FC amongst our rural folks underscore the need for continuous and better education of our mothers with regards to better child care. After the institution of the intervention measures, the proportion of respondents with adequate knowledge of FC increased to 70% and this increase was found to be statistically significant (*degedege* was caused by evil spirits) [21].

Inadequate first aid measures were the norm among the respondents. Prior to the intervention, over 90% of the mothers in this study used traditional medications including scarification marks during convulsive episodes on their children. This is in keeping with the works of Ofovwe and others where all their respondents in the rural area permitted the use of traditional medications [18]. A sizeable proportion (74%) of the mothers tried to pry the clenched teeth of the convulsing child apart and inserted spoon into the mouth, a practice which is meant to prevent the child from biting the tongue. This practice has been widely reported from similar studies [14, 20]. A few of the mothers (24%) took their children to the hospital without first aid during episodes of FC which is in consonance with findings from Taiwan and Libya [14, 22].

In this study, only a few of the mothers (28%) carried out tepid sponging of their children during episodes of FC. Similar observations were made in the study of Zeglam et al [22] where a few of their respondents (6%) carried out tepid sponging. However, in other studies, most of the mothers did nothing to the child at home before taking to the hospital [19, 20].

After the intervention, there was improvement in the home practices of the mothers with regards to FC and these changes were statistically significant [1, 8, 14, 22, 23].

## Conclusion

Findings from the study at baseline showed that the level of knowledge and home management of the mothers with respect to FC was grossly inadequate which may not be unconnected with the low literacy level of the study subjects. However, following the intervention, there was appreciable improvement in the knowledge scores and home management of FC. The use of community members in sensitization and education of the women folk has proved effective as they have been found to be sensitive to the cultural milieu of their communities.
